# TRAF6 Silencing Attenuates Multiple Myeloma Cell Adhesion to Bone Marrow Stromal Cells

**DOI:** 10.3390/ijms20030702

**Published:** 2019-02-06

**Authors:** Jonathan J. Morgan, Roisin M. McAvera, Lisa J. Crawford

**Affiliations:** Centre for Cancer Research and Cell Biology, Queen’s University Belfast, Belfast BT9 7BL, UK; jmorgan30@qub.ac.uk (J.J.M.); rmcavera01@qub.ac.uk (R.M.M.)

**Keywords:** Multiple Myeloma, TRAF6, BMSCs

## Abstract

The bone marrow (BM) microenvironment plays an important role in supporting proliferation, survival and drug resistance of Multiple Myeloma (MM) cells. MM cells adhere to bone marrow stromal cells leading to the activation of tumour-promoting signaling pathways. Activation of the NFκB pathway, in particular, is central to the pathogenesis of MM. Tumour necrosis factor receptor-associated factor 6 (TRAF6) is a key mediator of NFκB activation and has previously been highlighted as a potential therapeutic target in MM. Here, we demonstrate that adherence of MM cell lines to stromal cells results in a reciprocal increase in TRAF6 expression. Knockdown of TRAF6 expression attenuates the ability of MM cells to bind to stromal cells and this is associated with a decrease in NFκB-induced expression of the adhesion molecules ICAM1 and VCAM1. Finally, we show that knockdown of TRAF6 sensitizes MM cells to treatment with bortezomib when co-cultured with stromal cells. Inhibiting TRAF6 represents a promising strategy to target MM cells in the BM microenvironment.

## 1. Introduction

Multiple Myeloma (MM) is characterised by the proliferation of malignant plasma cells in the bone marrow, resulting in the excessive production of monoclonal protein in the serum and urine. MM manifests clinically with osteolytic bone disease, anaemia, immunodeficiency and renal insufficiency. Over the past 15 years, response rates and overall survival have significantly improved with the introduction of proteasome inhibitors [1], immunomodulatory agents [2], and more recently monoclonal antibodies [3], to the clinic. However, despite these improvements MM largely remains an incurable disease and acquired drug resistance and disease relapse present a clinical challenge [4,5].

The bone marrow (BM) microenvironment comprising bone marrow stromal cells (BMSCs), immune cells, osteoclasts, osteoblasts and extracellular matrix, plays a critical role in disease progression. MM cells home to and adhere to BMSCs, leading to the production of tumour-promoting cytokines and the activation of key signalling pathways, such as nuclear factor kappa B (NFκB), in both MM cells and BMSCs [6,7]. This interaction promotes MM cell proliferation, migration, survival and drug resistance. Through the upregulation of interleukin (IL)-6 and receptor activator of NFκB ligand (RANKL), BMSC–MM interactions also promote the differentiation and proliferation of osteoclasts which lead to bone resorption and further promote the growth of MM cells [8,9], forming a vicious cycle of bone destruction and MM cell proliferation. It is important, therefore, to develop novel therapeutic approaches which act not only on MM cells but also on the protective microenvironment.

Tumour Necrosis Factor Receptor-Associated Factor 6 (TRAF6) is an E3 ligase that functions primarily to activate downstream signalling cascades in a degradation-independent manner [10]. Upon ligand binding, TRAF6 is recruited to receptor complexes and catalyses K63-linked polyubiquitination on itself and its target proteins [11]. TRAF6-mediated polyubiquitination initiates signalling cascades that lead to the activation of NFκB, MAPK and PI3K [12,13,14]. CD40L, IL-1, IL-17 and RANKL, all of which are upregulated in the BM microenvironment, can bind to TRAF6 receptors and activate downstream signalling [15,16,17,18]. It is not surprising therefore, that upregulated TRAF6 expression is reported in MM [19]. Furthermore, it has previously been demonstrated that TRAF6 silencing inhibits MM cell proliferation in cell lines and primary cells and inhibits osteoclast formation and bone resorption [19,20]. In this study, we sought to investigate the effect of TRAF6 silencing on MM–BMSC interactions.

## 2. Materials and Methods

### 2.1. Cell Lines and Reagents

U266, KMS-11, HS-5 and Phoenix GP cells were cultured in a humidified incubator at 37 °C and 5% CO_2_ in RPMI 1640 medium supplemented with 10% foetal bovine serum (FBS), 100 U/mL penicillin and 100 µg/mL streptomycin (Fisher Scientific, Loughborough, UK). Bortezomib was purchased from Selleckchem (Absource Diagnosticsm Munchen, Germany), reconstituted in DMSO and stored at −20 °C.

### 2.2. Retroviral Transfection

Retroviral particles expressing an shRNA against TRAF6 were generated by transfection of a pRSC vector together with pMD2.G into Phoenix GP cells. The shRNA sequence is shown below. Cells infected with a vector expressing non-targeting control (NTC) shRNA were used as controls. Cells were selected for in 0.5 µg/mL puromycin (Sigma-Aldrich, Dorset, UK) 48 h post-transduction.

Sequence: shTRAF6 GAGAACACCCAGTCACACA

### 2.3. Stromal Cell Co-Culture

Stromal cell co-cultures were set up using either the human stromal cell line HS-5 or BMSCs from MM patients. Patient BMSCs were isolated from BM aspirates as previously described [21]. MM cell lines or stromal cells were cultured either alone or together at a 1:5 (BMSC/MM) ratio and cell proliferation was measured using CyQUANT^®^ Direct cell proliferation assay, as per the manufacturer’s instructions (ThermoFisher Scientific, Loughborough, UK). If required, separation of myeloma cells from the HS-5 stromal cells was performed by selection with anti-CD138 magnetic cell separation micro beads using the AutoMACS system (Miltenyi Biotech, Bergisch Gladbach, Germany) according to the manufacturer’s instructions.

### 2.4. Adhesion Assay

HS-5 stromal cells and stromal cells obtained from MM patients were seeded at 1 × 10^4^ in 100 μL per well in a flat-bottom 96-well optical plate and cultured overnight to establish an adherent monolayer. MM cell lines were incubated with Calcein-AM (Abcam, Cambridge, UK) for 1 h, added to stromal cells at a concentration of 5 × 10^5^/mL and allowed to adhere for 2 h. Plates were gently inverted to remove media and washed 3 times with RPMI 1640 medium to remove non-adherent cells. Fluorescence was measured at excitation 485 nm/emission 520 nm using a Tecan Genios microplate reader.

### 2.5. Real-Time PCR

Total RNA was extracted from cells using ReliaPrep™ RNA Cell Miniprep System (Promega, Wisconsin, UK). An amount of 500 ng RNA was converted to cDNA using MMLV (Invitrogen, Carlsbad, CA, USA) according to the manufacturer’s instructions. The primers for TRAF6, VCAM1, ICAM1 and 18S were designed against Genbank published sequences in association with Primer Express (Applied Biosystems) and were obtained from Eurofins MWG Operon (Huntsville, AL, USA). Amplification for primers was performed using the FastStart Universal SYBR Green master mix (Roche Applied Science, Mannheim, Germany). Reactions were performed on a 7900HT fast Real-Time PCR system using ABI sequence detection software v2.3 (Applied Biosystems, Warrington, UK). Values were normalized to the endogenous 18S rRNA control and the relative mRNA fold changes were quantified using the 2^−ΔΔ*C*t^ method.

Primers:

ICAM1

Forward: CAGGGAACTGGTCAGGAACC

Reverse: ATCCTCAGCCCTAAGGAGCA

VCAM1

Forward: CGTCTTGGTCAGCCCTTCCT

Reverse: ACATTCATATACTCCCGCATCCTTC

TRAF6

Forward: TTCAGTACTTTTGGTTGCCATGA

Reverse: TGTGACTGGGTGTTCTCTTGTAGGT

### 2.6. Western Blotting

Cells were harvested and lysed in radioimmunoprecipitation (RIPA) buffer containing protease and phosphatase inhibitors. An amount of 20 µg of protein for each sample was denatured in LDS sample buffer (Invitrogen Ltd., Paisley, UK) at 95 °C for 5 min and resolved by SDS-PAGE on 10% Bis-Tris gels (Invitrogen Ltd., Paisley, UK). The protein was then transferred to a polyvinylidene fluoride (PVDF) membrane. Immunoblotting was carried out using antibodies against GAPDH (Abcam, Cambridge, UK), TRAF6, Total/p-IKKα/β, Pan-Actin, Total/p- TRAF6, Total/p-IKα, Total/p-p65 (Cell Signaling Technology, Hertfordshire, UK) and secondary antibodies anti-mouse and anti-rabbit (DAKO, Cambridgeshire, UK). Blots were probed with WesternBright™ ECL horseradish peroxidase (HRP) substrate (Advansta, Labtech, East Sussex, UK) for visualisation of protein.

### 2.7. Statistical Analysis

Data was processed using Prism v.6 (GraphPad Software, San Diego, CA, USA). Student’s *t*-test was used to derive statistical significance.

## 3. Results

### 3.1. TRAF6 Expression Is Enhanced Upon Adherence of MM Cells to BMSCs

**Figure 1:**
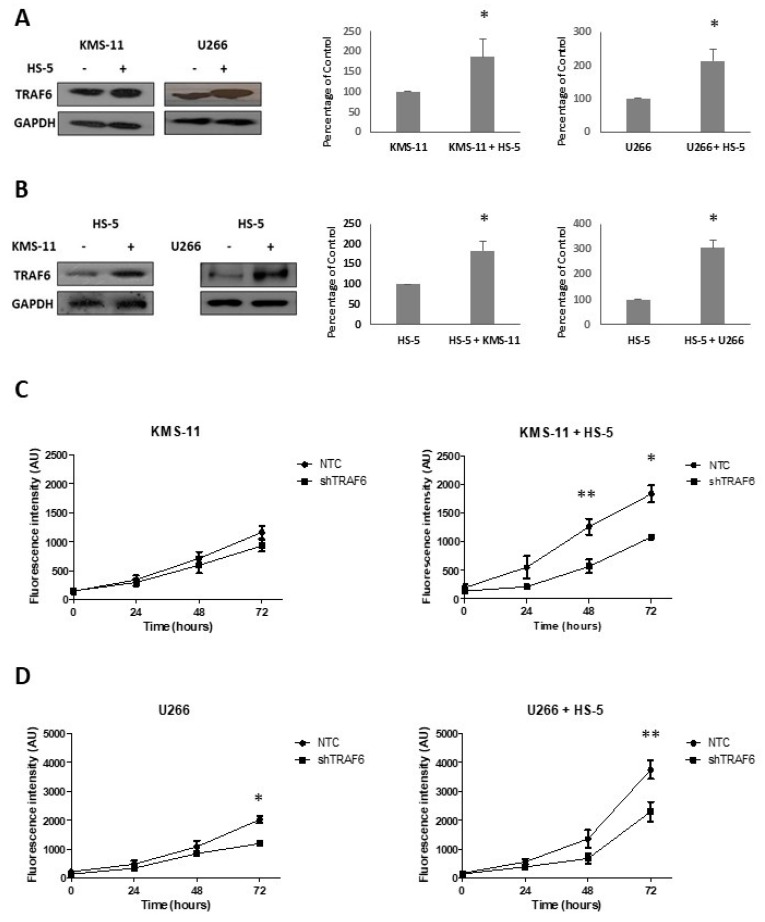
Tumour necrosis factor receptor-associated factor 6 (TRAF6) expression is enhanced in bone marrow stromal cell (BMSC) co-cultures: (**A**) TRAF6 protein expression in KMS-11 and U266 cells cultured on their own or in co-culture with HS-5 cells; optical density normalized to GAPDH and expressed as a percentage of KMS-11 or U266 cells cultured alone (*n* = 3). (**B**) TRAF6 protein expression in HS-5 cells cultured on their own or in co-cultures with KMS-11 or U266 cells; optical density normalized to GAPDH and expressed as a percentage of HS-5 cells cultured alone (*n* = 3). (**C**) Proliferation of KMS-11 cells transduced with non-targeting control (NTC) shRNA or shRNA targeting TRAF6 (shTRAF6), cultured in isolation (left panel) or in co-culture with HS-5 cells (right panel), *n* = 4; (**D**) Proliferation of U266 cells transduced with NTC shRNA or shRNA targeting TRAF6, cultured in isolation (left panel) or in co-culture with HS-5 cells (right panel), *n* = 4. * *p* ≤ 0.05, ** *p* ≤ 0.01.

BMSCs play an important role in the survival of MM cells and contribute to cell adhesion-mediated drug resistance. Huang and colleagues recently demonstrated that BMSCs induce the expression of TRAF6 in MM cell lines [22]. To investigate whether TRAF6 expression is similarly affected in BMSCs, we co-cultured KMS-11 and U266 cell lines with the human stromal cell line HS-5. After 24 h, MM and HS-5 co-cultures were separated by CD138+ selection of MM cell lines and TRAF6 protein expression was evaluated in cell lines, HS-5 cells, and matched cells that had been cultured in isolation. TRAF6 protein levels significantly increased both in MM cell lines (Figure 1A, *p* ≤ 0.05) and BMSCs (Figure 1B *p* ≤ 0.02) that had been co-cultured compared to cells that had been grown in single cultures, suggesting that TRAF6 is activated by BMSC–MM interactions. We next looked at the effect of TRAF6 silencing on the proliferation of MM cell lines cultured in the presence and absence of HS-5 cells. In general, TRAF6 knockdown cells (shTRAF6) grew significantly more slowly than their control counterparts (NTC—non-targeting control) (Figure 1C,D; *p* ≤ 0.04, 72 h; not significant for KMS-11 single cultures). Co-culture with HS-5 cells increased the growth of both control and TRAF6 knockdown cell lines, however, proliferation of both KMS-11 and U266 TRAF6 knockdown cells was most significantly reduced in stromal cell co-cultures compared to those grown in the absence of HS-5 cells (*p* ≤ 0.04). To investigate the upstream molecules important for TRAF6 activation in MM cells, we looked at the effect of blocking CD40 and RANKL activation of TRAF6 using inhibitory peptides, however, inhibition of either of these interactions alone had no significant effect on MM cell growth (data not shown).

### 3.2. TRAF6 Knockdown Impairs Adhesion to BMSCs

**Figure 2:**
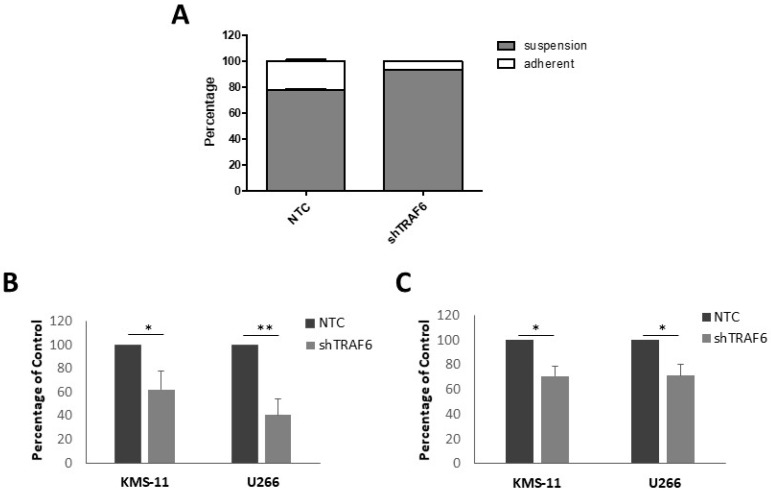
TRAF6 knockdown disrupts adhesion to BMSCs: (**A**) Proportion of suspension and adherent cells in KMS-11 TRAF6 knockdown cells (shTRAF6) compared to non-targeting control (NTC) cells; (**B**) Effect of TRAF6 knockdown on the ability of KMS-11 and U266 cells to adhere to HS-5 cells; (**C**) Effect of TRAF6 knockdown on the ability of KMS-11 and U266 cells to adhere to BMSCs from MM patients. The data is presented as mean (± st dev) of three independent experiments. * *p* ≤ 0.05, ** *p* ≤ 0.01.

Adhesion of MM cells to BMSCs stimulates NFκB transcription of adhesion molecules [23]. As TRAF6 is a key modulator of NFκB activation, we speculated that TRAF6 silencing could alter the adherent properties of MM cells. KMS-11 is a semi-adherent cell line that grows in tissue culture flasks as a mixture of adherent and non-adherent cells. Knockdown of TRAF6 in KMS-11 cells resulted in a significant decrease in the proportion of adherent cells compared to control cells (Figure 2A, *p* = 0.02). We next investigated the ability of TRAF6 knockdown cells to adhere to BMSCs using a fluorescence-based adhesion assay. KMS-11 and U266 cells were labelled with Calcein-AM and adhesion to both HS-5 and BMSCs from MM patients was measured. TRAF6 knockdown cells exhibited a significant reduction in adhesion to both HS-5 and patient BMSCs (Figure 2B,C, *p* ≤ 0.05).

### 3.3. Knockdown of TRAF6 Inhibits NFκB Signalling

**Figure 3:**
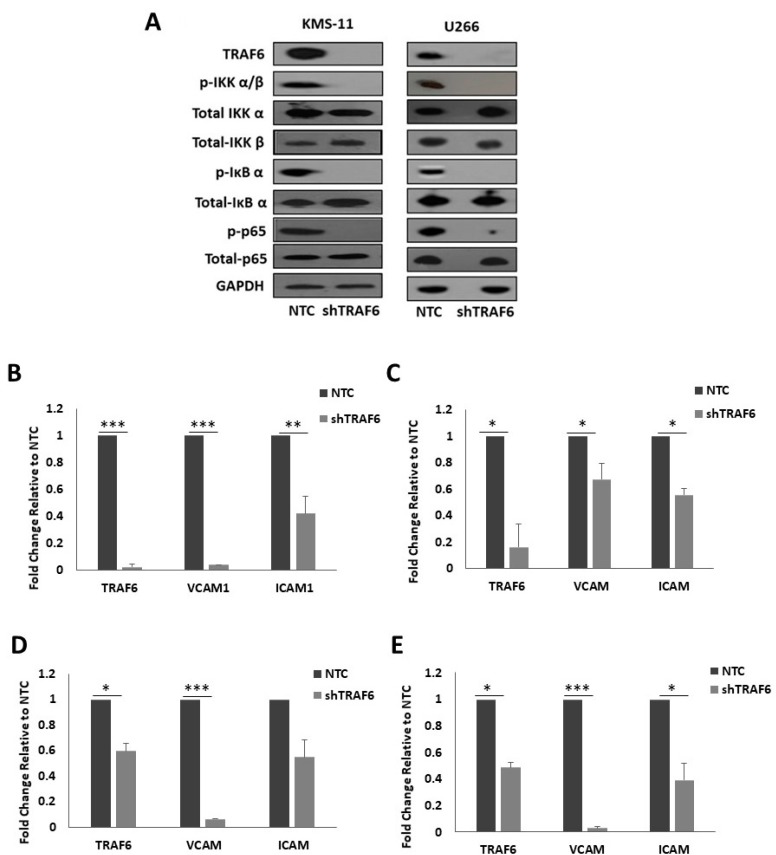
TRAF6 knockdown inhibits NFκB signaling and reduces NFκB-dependent transcription of adhesion molecules: (**A**) Protein expression of TRAF6 and key proteins in the activation of NFκB signaling. q-PCR analysis of TRAF6, VCAM1 and ICAM1 gene expression in (**B**) KMS-11 TRAF6 knockdown (shTRAF6) and non-targeting control (NTC) cells, (**C**) U266 shTRAF6 and NTC cells, (**D**) HS-5 cells co-cultured with KMS-11 NTC or shTRAF6 cells and (**E**) HS-5 cells co-cultured with U266 NTC or shTRAF6 cells. The data is presented as mean (± st. dev) of three independent experiments. * *p* ≤ 0.05, ** *p* ≤ 0.01, *** *p* ≤ 0.001.

TRAF6 has previously been implicated in the regulation of NFκB activation in MM cells [19,20,22]. As NFκB is known to promote the expression of a number of adhesion molecules, we looked at the effect of TRAF6 silencing on NFκB pathway activation in MM cells and on the expression of NFκB target genes, intracellular adhesion molecule 1 (ICAM1) and vascular cell adhesion molecule 1 (VCAM1). Knockdown of TRAF6 in both cell lines led to a striking decrease in the phosphorylation of inhibitor of NFκB kinase α/β (IKK α/β), IκB α and p65 (Figure 3A) and this was accompanied with a significant decrease in the mRNA expression of the adhesion molecules ICAM1 and VCAM1 (Figure 3B,C, *p* ≤ 0.04). Furthermore, the expression of TRAF6, and of ICAM1 and VCAM1, was also decreased in HS-5 cells that were co-cultured with shTRAF6 KMS-11 and U266 cells (Figure 3D,E, *p* ≤ 0.03).

### 3.4. TRAF6 Knockdown Augments the Effect of Bortezomib in Stromal Co-Cultures

**Figure 4:**
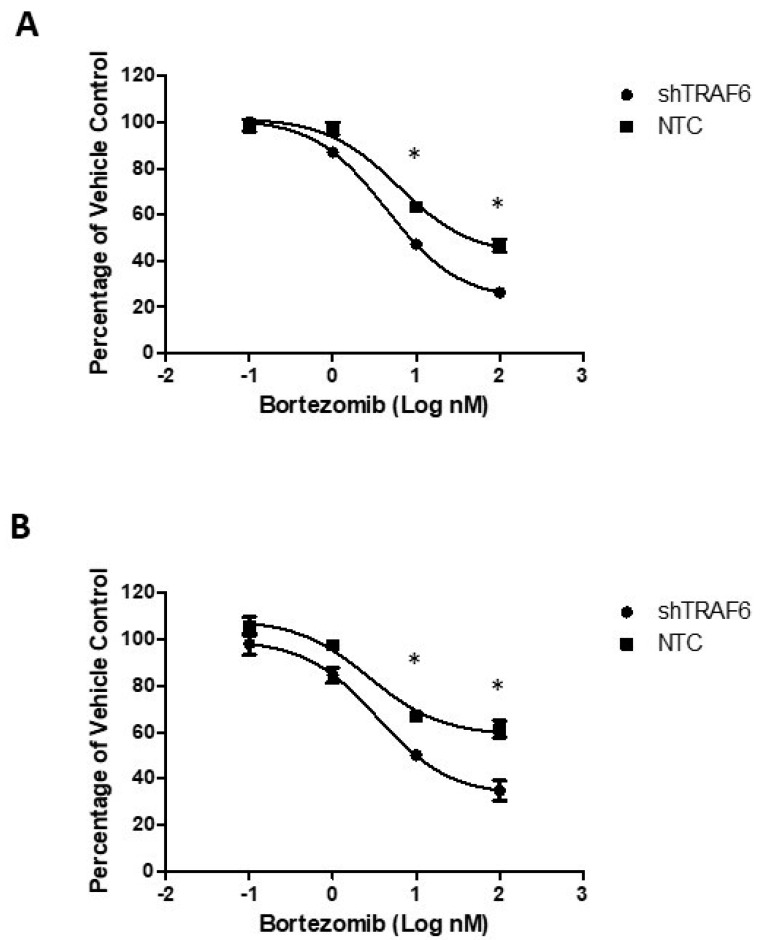
TRAF6 knockdown enhances the efficacy of bortezomib in MM–BMSC co-cultures: (**A**) Dose response of KMS-11 NTC and shTRAF6 cells co-cultured with HS-5; (**B**) Dose response of U266 NTC and shTRAF6 cells co-cultured with HS-5. The data is presented as mean (±st. dev) of three independent experiments, * *p* ≤ 0.05.

Co-culture of MM cell lines with stromal cells attenuates the sensitivity of MM cells to therapeutic agents [24]. We evaluated the effect of the proteasome inhibitor bortezomib on TRAF6 knockdown cells compared to control cells when co-cultured with HS-5 cells. Knockdown of TRAF6 in combination with bortezomib resulted in a significant decrease in viability of both KMS-11 (Figure 4A; *p* ≤ 0.33) and U266 cells (Figure 4B; *p* ≤ 0.24), compared to the effect of bortezomib in control cells, suggesting that targeting of this E3 ligase could help to overcome stromal cell-induced protection against therapeutic agents.

## 4. Discussion

Adherence of MM cells to BMSCs activates signalling pathways that promote the growth, survival and drug resistance of MM cells. Activation of the NFκB pathway, in particular, is central to the pathogenesis of MM [25]. The E3 ligase TRAF6 is a key mediator of NFκB activation and has previously been highlighted as a potential therapeutic target in MM [19,20,22].

A recent study found that BMSCs can induce TRAF6 expression in MM cells [22]. In accordance with this, we demonstrate the increased expression of TRAF6 when MM cells are co-cultured with BMSCs. We also found that there is a reciprocal increase in TRAF6 expression in stromal cells upon adherence of MM cell lines. Conversely, we observe a decrease in TRAF6 expression in BMSCs co-cultured with TRAF6 knockdown MM cell lines compared to control cells. As the interaction of MM and BMSCs activates NFκB signalling in both the malignant plasma cells and in stromal cells [26], this suggests that TRAF6 may mediate NFκB signalling in both cell types. Consistent with a number of studies [19,22], we found that knockdown of TRAF6 reduced the proliferation of MM cell lines and we demonstrate that this effect was most pronounced when MM cell lines were grown in co-culture with stromal cells. To identify the upstream molecules important for TRAF6 activation in MM cells, we used inhibitory peptides to disrupt TRAF6 interactions with either CD40 or RANKL. Inhibition of TRAF6-CD40 or TRAF6-RANKL did not significantly affect the growth of MM cell lines cultured alone or in co-culture with stromal cells, suggesting that either other upstream signalling factors may be more important or that a combination of factors is involved in TRAF6 signalling pathways in MM.

NFκB is comprised of heterodimers of the Rel family of transcription factors that commonly exists as a dimer of p65 and p50 subunits. Inactive NFκB is sequestered in the cytoplasm bound to its inhibitor IκBα and can be activated through a diverse range of stimuli [27]. TRAF6-mediated activation of NFκB results from K63-linked polyubiquitin of TRAF6 itself and other TRAF6 substrates, leading to recruitment of the TAK1 kinase and subsequent phosphorylation and activation of the IKK complex (composed of IKKα and IKKβ) [11]. IKK α/β, in turn, phosphorylates IκBα, promoting its degradation through the proteasome and freeing NFκB to locate to the nucleus and initiate transcription. Here, we show that knockdown of TRAF6 blocks the phosphorylation of IKK in MM cells lines which subsequently blocks phosphorylation of IκBα and p65.

NFκB induces the expression of adhesion molecules VCAM1 and ICAM1 on both MM and BMSCs [23]. As TRAF6 knockdown inhibits NFκB signalling in MM, we hypothesised that it would also affect the adhesive properties of MM cells. Looking first at the effect of TRAF6 knockdown on semi-adherent cell line KMS-11, we found a significant reduction in the proportion of adherent cells in TRAF6 knockdown cells. Furthermore, we demonstrated that both KMS-11 and U266 cells displayed a reduced ability to adhere to a stromal cell line and to BMSCs isolated from MM patients. The associated decrease in the expression of NFκB target genes ICAM1 and VCAM1 in both MM cells and BMSCs, suggests that TRAF6-mediated activation of NFκB signalling contributes to their upregulation and promotes the adherence of MM cells to BMSCs. The elevated expression of adhesion molecules, ICAM1 and VCAM1, is associated with disease progression in MM [28]. The reduction in the expression of these molecules on both MM cells and BMSCs following TRAF6 knockdown lends further weight to the potential of TRAF6 as a therapeutic target in MM.

Silencing of TRAF6 has been reported to enhance the effect of bortezomib in MM [19] and acute myeloid leukaemia/myelodysplastic syndrome [29]. Interactions with BMSCs are well documented to protect MM cells from drug-induced apoptosis and to contribute to acquired resistance to therapies [30,31,32]. With this is mind, we investigated the effect of TRAF6 knockdown in combination with bortezomib in MM cell lines co-cultured with stromal cells. TRAF6 knockdown similarly enhanced the effect of bortezomib in stromal cell co-cultures, suggesting that the combination of TRAF6 inhibition with bortezomib could effectively target MM cells within the bone marrow microenvironment.

In MM, adhesion to BMSCs plays an important role in the growth and survival of tumour cells. This study revealed that TRAF6 is important in mediating interactions and adherence to BMSCs. These findings, taken together with previous studies reporting that TRAF6 inhibition reduced osteoclast formation [19], demonstrate that inhibition of TRAF6 represents a promising approach to target not only MM cells but also their BM microenvironment.

## Abbreviations

BM	Bone marrow
BMSC	Bone marrow stromal cell
ICAM1	Intracellular adhesion molecule 1
MM	Multiple Myeloma
NFκB	Nuclear factor kappa B
TRAF6	Tumour necrosis factor receptor-associated factor 6
VCAM1	Vascular adhesion molecule 1

## References

[1] Guerrero-Garcia T.A., Gandolfi S., Laubach J.P., Hideshima T., Chauhan D., Mitsiades C., Anderson K.C., Richardson P.G. (2018). The Power of Proteasome Inhibition in Multiple Myeloma. Expert Rev. Proteom..

[2] Holstein S.A., McCarthy P.L. (2017). Immunomodulatory Drugs in Multiple Myeloma: Mechanisms of Action and Clinical Experience. Drugs.

[3] Varga C., Maglio M., Ghobrial I.M., Richardson P.G. (2018). Current use of Monoclonal Antibodies in the Treatment of Multiple Myeloma. Br. J. Haematol..

[4] Guang M.H.Z., McCann A., Bianchi G., Zhang L., Dowling P., Bazou D., O’Gorman P., Anderson K.C. (2018). Overcoming Multiple Myeloma Drug Resistance in the Era of Cancer ‘Omics’. Leuk. Lymphoma.

[5] Robak P., Drozdz I., Szemraj J., Robak T. (2018). Drug Resistance in Multiple Myeloma. Cancer Treat. Rev..

[6] Urashima M., Chen B.P., Chen S., Pinkus G.S., Bronson R.T., Dedera D.A., Hoshi Y., Teoh G., Ogata A., Treon S.P. (1997). The Development of a Model for the Homing of Multiple Myeloma Cells to Human Bone Marrow. Blood.

[7] Chauhan D., Uchiyama H., Akbarali Y., Urashima M., Yamamoto K., Libermann T.A., Anderson K.C. (1996). Multiple Myeloma Cell Adhesion-Induced Interleukin-6 Expression in Bone Marrow Stromal Cells Involves Activation of NF-Kappa B. Blood.

[8] Michigami T., Shimizu N., Williams P.J., Niewolna M., Dallas S.L., Mundy G.R., Yoneda T. (2000). Cell-Cell Contact between Marrow Stromal Cells and Myeloma Cells Via VCAM-1 and Alpha(4)Beta(1)-Integrin Enhances Production of Osteoclast-Stimulating Activity. Blood.

[9] Sezer O., Heider U., Zavrski I., Kuhne C.A., Hofbauer L.C. (2003). RANK Ligand and Osteoprotegerin in Myeloma Bone Disease. Blood.

[10] Inoue J., Gohda J., Akiyama T. (2007). Characteristics and Biological Functions of TRAF6. Adv. Exp. Med. Biol..

[11] Deng L., Wang C., Spencer E., Yang L., Braun A., You J., Slaughter C., Pickart C., Chen Z.J. (2000). Activation of the IkappaB Kinase Complex by TRAF6 Requires a Dimeric Ubiquitin-Conjugating Enzyme Complex and a Unique Polyubiquitin Chain. Cell.

[12] Fang J., Muto T., Kleppe M., Bolanos L.C., Hueneman K.M., Walker C.S., Sampson L., Wellendorf A.M., Chetal K., Choi K. (2018). TRAF6 Mediates Basal Activation of NF-kappaB Necessary for Hematopoietic Stem Cell Homeostasis. Cell Rep..

[13] Xiao F., Wang H., Fu X., Li Y., Wu Z. (2012). TRAF6 Promotes Myogenic Differentiation Via the TAK1/p38 Mitogen-Activated Protein Kinase and Akt Pathways. PLoS ONE.

[14] Hamidi A., Song J., Thakur N., Itoh S., Marcusson A., Bergh A., Heldin C.H., Landstrom M. (2017). TGF-Beta Promotes PI3K-AKT Signaling and Prostate Cancer Cell Migration through the TRAF6-Mediated Ubiquitylation of p85alpha. Sci. Signal..

[15] Hostager B.S. (2007). Roles of TRAF6 in CD40 Signaling. Immunol. Res..

[16] Cao Z., Xiong J., Takeuchi M., Kurama T., Goeddel D.V. (1996). TRAF6 is a Signal Transducer for Interleukin-1. Nature.

[17] Rong Z., Cheng L., Ren Y., Li Z., Li Y., Li X., Li H., Fu X.Y., Chang Z. (2007). Interleukin-17F Signaling Requires Ubiquitination of Interleukin-17 Receptor Via TRAF6. Cell. Signal..

[18] Mizukami J., Takaesu G., Akatsuka H., Sakurai H., Ninomiya-Tsuji J., Matsumoto K., Sakurai N. (2002). Receptor Activator of NF-kappaB Ligand (RANKL) Activates TAK1 Mitogen-Activated Protein Kinase Kinase Kinase through a Signaling Complex Containing RANK, TAB2, and TRAF6. Mol. Cell. Biol..

[19] Chen H., Li M., Sanchez E., Wang C.S., Lee T., Soof C.M., Casas C.E., Cao J., Xie C., Udd K.A. (2017). Combined TRAF6 Targeting and Proteasome Blockade has Anti-Myeloma and Anti-Bone Resorptive Effects. Mol. Cancer. Res..

[20] Chen H., Li M., Campbell R.A., Burkhardt K., Zhu D., Li S.G., Lee H.J., Wang C., Zeng Z., Gordon M.S. (2006). Interference with Nuclear Factor Kappa B and c-Jun NH2-Terminal Kinase Signaling by TRAF6C Small Interfering RNA Inhibits Myeloma Cell Proliferation and Enhances Apoptosis. Oncogene.

[21] Crawford L.J., Anderson G., Johnston C.K., Irvine A.E. (2016). Identification of the APC/C Co-Factor FZR1 as a Novel Therapeutic Target for Multiple Myeloma. Oncotarget.

[22] Huang H., Sun Z., Wang X., Liu X., Na W., Xu R., Ding R., Liu H. (2017). The Effect of Marrow Stromal Cells on TRAF6 Expression Levels in Myeloma Cells. Oncol. Lett..

[23] Gupta D., Treon S.P., Shima Y., Hideshima T., Podar K., Tai Y.T., Lin B., Lentzsch S., Davies F.E., Chauhan D. (2001). Adherence of Multiple Myeloma Cells to Bone Marrow Stromal Cells Upregulates Vascular Endothelial Growth Factor Secretion: Therapeutic Applications. Leukemia.

[24] Misund K., Baranowska K.A., Holien T., Rampa C., Klein D.C., Borset M., Waage A., Sundan A. (2013). A Method for Measurement of Drug Sensitivity of Myeloma Cells Co-Cultured with Bone Marrow Stromal Cells. J. Biomol. Screen..

[25] Roy P., Sarkar U.A., Basak S. (2018). The NF-kappaB Activating Pathways in Multiple Myeloma. Biomedicines.

[26] Podar K., Richardson P.G., Hideshima T., Chauhan D., Anderson K.C. (2007). The Malignant Clone and the Bone-Marrow Environment. Best Pract. Res. Clin. Haematol..

[27] Christian F., Smith E.L., Carmody R.J. (2016). The Regulation of NF-kappaB Subunits by Phosphorylation. Cells.

[28] Terpos E., Migkou M., Christoulas D., Gavriatopoulou M., Eleutherakis-Papaiakovou E., Kanellias N., Iakovaki M., Panagiotidis I., Ziogas D.C., Fotiou D. (2016). Increased Circulating VCAM-1 Correlates with Advanced Disease and Poor Survival in Patients with Multiple Myeloma: Reduction by Post-Bortezomib and Lenalidomide Treatment. Blood Cancer. J..

[29] Fang J., Rhyasen G., Bolanos L., Rasch C., Varney M., Wunderlich M., Goyama S., Jansen G., Cloos J., Rigolino C. (2012). Cytotoxic Effects of Bortezomib in Myelodysplastic syndrome/acute Myeloid Leukemia Depend on Autophagy-Mediated Lysosomal Degradation of TRAF6 and Repression of PSMA1. Blood.

[30] Murray M.Y., Auger M.J., Bowles K.M. (2014). Overcoming Bortezomib Resistance in Multiple Myeloma. Biochem. Soc. Trans..

[31] Manni S., Carrino M., Semenzato G., Piazza F. (2018). Old and Young Actors Playing Novel Roles in the Drama of Multiple Myeloma Bone Marrow Microenvironment Dependent Drug Resistance. Int. J. Mol. Sci..

[32] Di Marzo L., Desantis V., Solimando A.G., Ruggieri S., Annese T., Nico B., Fumarulo R., Vacca A., Frassanito M.A. (2016). Microenvironment Drug Resistance in Multiple Myeloma: Emerging New Players. Oncotarget.

